# Supplementary figures and images for: Postharvest bacterial succession on cut flowers and vase water
Source: PLoS One. 2023 Oct 10;18(10):e0292537. doi: 10.1371/journal.pone.0292537 (PMC10564175; doi:10.1371/journal.pone.0292537)

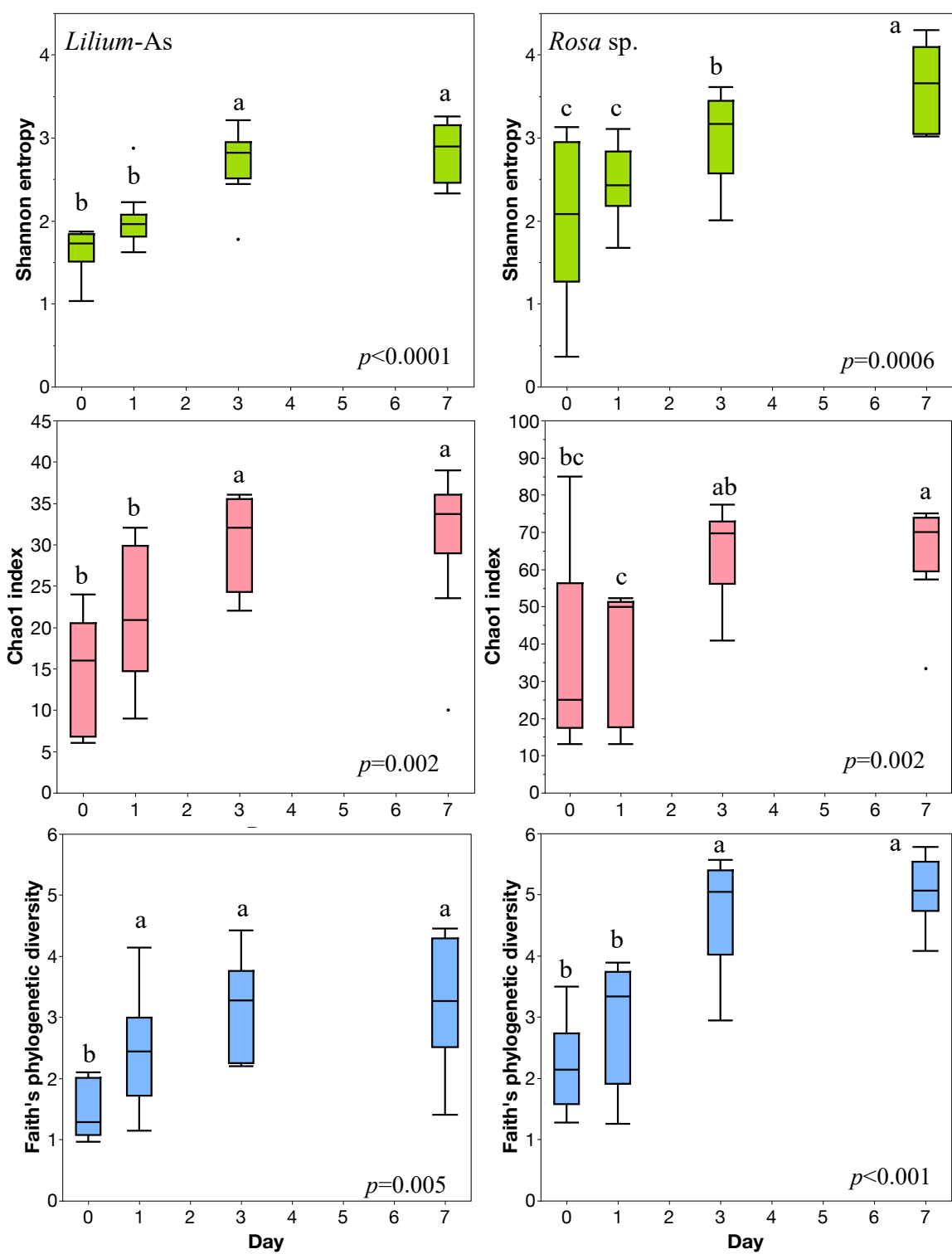

Supplement: S1 Fig — (PDF) [file pone.0292537.s001.pdf]
