# Supplementary material for: Postharvest bacterial succession on cut flowers and vase water
Source: PLoS One. 2023 Oct 10;18(10):e0292537. doi: 10.1371/journal.pone.0292537 (PMC10564175; doi:10.1371/journal.pone.0292537)
Supplement: S1 Table — (PDF) [file pone.0292537.s003.pdf]

S1 Table. The comparison of principal components in microbial communities from different flower source.

Flower Source	**PC1**	**PC2**
Retailer (Wegmans supermarket)	1.937 [z]	0.326
Grower (Cornell greenhouse)	-5.072	-1.286
***p*-value** [y]	<0.0001	0.0395

[z] Data are average principal component value of all samples per flower source.
[y] Data in columns were tested with two-tailed t test at significance of p-value < 0.05.
