# Supplementary material for: Postharvest bacterial succession on cut flowers and vase water
Source: PLoS One. 2023 Oct 10;18(10):e0292537. doi: 10.1371/journal.pone.0292537 (PMC10564175; doi:10.1371/journal.pone.0292537)
Supplement: S2 Table — (PDF) [file pone.0292537.s004.pdf]

S2 Table. Identified bacteria in postharvest research of cut flowers.

Floral crops	Sample type	Identified bacteria taxa	Reference
Gerbera jamesonii	Vase water	*Pseudomonas aeruginosa Enterobacteriaceae, Pseudomonas veronii, Pseudomonas spp., Delftia spp., Agrobacterium spp., Sphingobacterium multivorum, Acinetobacter johnsonii, and Clostridiaceae.*	[12] [21]
	Scape of flowers	*Acinetobacter*, *Bacillus* and *Pantoea*	[7]
Dianthus caryophyllus **'Scania'**	Vase water	*Enterobacter agglomerans*, *Alcaligenes spp.*, and *Acinetobacter calcoaceticus*	[19]
Rosa **'Sonia'**	Vase water	*Pseudomonas spp. (P. aeruginosa, P. cepacian, P. maltophilia, P. putida, P. stutzeri, and P. vesicularis)*	[19]
Zinnia elegans	Vase water	*Pseudomonas fulva, Pseudomonas marginalis Serratia ficaria*, *Rhizobium radiobacter*, *Chryseobacterium spp.*, *Pantoea ananatis*, *Bacillus pumilus*, *Chryseobacterium daejeonense*, and *Brevundimonas spp.*	[13]
