# Supplementary material for: Postharvest bacterial succession on cut flowers and vase water
Source: PLoS One. 2023 Oct 10;18(10):e0292537. doi: 10.1371/journal.pone.0292537 (PMC10564175; doi:10.1371/journal.pone.0292537)
Supplement: Attachment — Submitted filename: Rebuttal letter-Response to Reviewers.docx [file pone.0292537.s005.docx]

**Reviewers' comments:**
Reviewer #1: The authors studied the dynamic succession of microbial communities in the vase ecosystem of different cut flower varieties at the 16S rRNA gene level, providing clues for managing cut flowers as well as improving the longevity of cut flower vases. However, there are some issues in manuscript that need to be further addressed, as follows:
1. The authors study the succession of microbial communities in cut flower vase water, which I think should be presented in the title. However, the title “Microbial succession on cut flowers” does not reflect the study fully.

**Ans: Done. Change the title as follows:**

**Postharvest bacterial succession on cut flowers and vase water.**

2.“…each vase holding flowers on day 1, 3, and 7, and the control vase on day 0, 1, 3, 7.” Sampling time should be included here.

**Ans: Done. Added in Line 134: The sampling time of day 0 was October 31, 2020.**

3. In the results section, it is suggested that the subtitles should be added for descriptions.

**Ans: Done. Subtitles added as following texts:**

**Line189: The dynamics of chloroplast and mitochondrial small subunit (SSU) amplicons during the vase period.**

**Line214: The bacterial diversity of flowers and vase water by source and cultivar.**

**Line284: Community differences between sampling locations over time for *Lilium* ‘Sorbonne’ and *Gerbera jamesonii***

4. Line 162 of the manuscript mentions that "In all 4 flower types, the initial relative abundance of chloroplast sequences ranged from 69-82% of all amplicons." However, Fig 1a shows that the initial relative abundance of chloroplast in Lily-As ranges from 20-30%, which is not consistent with the text description. Please check the results section for the same mistakes.

**Ans: Done. Revised in Line 202-204 as following texts:**

**The initial relative abundance of chloroplast sequences ranged from 25-85% (Fig 1). Chloroplast relative abundance decreased significantly over seven days for all samples (*P*_t0 vs t7_ <0.001), yielding fold changes ranging from 3.6 to 32.8.**

5.I couldn’t see the SRA (sequence read archive) accession numbers for the samples.

**Ans: Done. All were uploaded in NCBI.**

6.The authors analyzed stem and water samples of Gerbera jamesonii in order to better understand changes in specific OTUs over time. For this reason, I wonder why Gerbera jamesonii was chosen? Can gerbera daisies represent the remaining three flowers?

**Ans: Done. Explained in Line 276 -282 as following texts:**

**There are two flower sources, *Lilium* ‘Sorbonne’ was grown in the greenhouse of Cornell University, and the other three flower crops were purchased from the local supermarket. *Gerbera jamesonii* is one of the major cut flowers in the globe. The gerbera flower scape surface is hairy, and the anatomical structure of the scape is dissimilar to the other flower types. Based on Table 2 and Fig 3, the further investigations of *Lilium* ‘Sorbonne’ and *Gerbera jasmonii* as our representative flowers are present in Figs 4 and 5.**

7.The Latin name of the bacterium should be italicized as it appears in Figures 6, 7, and 8.

**Ans: Done.**

8.Please keep the reference format consistent in the manuscript, and “Figure” or “Fig”, please use the same style in manuscript.

**Ans: Done.**

Reviewer #2: Dear authors
Overall, the this paper provides a comprehensive overview of a study on microbial succession on cut flowers and its impact on post harvest quality. However, there are some weaknesses in the text that can be addressed:

1- Lack of clarity in the objectives: The introduction section does not clearly state the specific objectives of the study. It should clearly define the research questions or hypotheses that the study aimed to address.

**Ans: Done. The hypothesis was emphasized in Line 98-100 as following texts:**

**The research hypothesis was that bacterial communities would change by flower source, type, and location (water vs. stem) over time.**


2- Inadequate background information: The text lacks a thorough literature review to establish the existing knowledge and research gaps in the field. Including more background information and citing relevant studies would strengthen the introduction.
**Ans: Done. The two references were added, [16] and [35]. Newly added texts in Line 94-95, and Line 508-515.**

3- Inconsistent use of terminology: The text inconsistently uses terms such as "microbes," "bacteria," and "microbial communities." Standardizing the terminology would improve the clarity and precision of the text.

**Ans: Done. We standardize the terms to “bacteria” and “bacterial communities”**

4- Inadequate discussion of results: The discussion section should provide a more in-depth analysis and interpretation of the results, linking them back to the research objectives and existing literature. It should also address any limitations or potential sources of bias in the study.
**Ans: Please read the newly added interpretation in Line 508-515, and Line531-536.**

5-Lack of conclusion: The text does not include a separate conclusion section summarizing the main findings of the study. Adding a concise conclusion would provide a clear ending to the research article.

**Ans: Done. Conclusion added in Line 537-540 as following texts:
In conclusion, the bacterial diversity varied by flower source and type, and we presented the bacterial succession in flower stems and vase water over time. This would be a critical foundation for further research on postharvest physiology and microbiology.**

Journal Requirements:

When submitting your revision, we need you to address these additional requirements.

1. Please ensure that your manuscript meets PLOS ONE's style requirements, including those for file naming. The PLOS ONE style templates can be found at
<https://journals.plos.org/plosone/s/file?id=wjVg/PLOSOne_formatting_sample_main_body.pdf> and
<https://journals.plos.org/plosone/s/file?id=ba62/PLOSOne_formatting_sample_title_authors_affiliations.pdf>

**Ans: Done.**

2. In your Data Availability statement, you have not specified where the minimal data set underlying the results described in your manuscript can be found. PLOS defines a study's minimal data set as the underlying data used to reach the conclusions drawn in the manuscript and any additional data required to replicate the reported study findings in their entirety. All PLOS journals require that the minimal data set be made fully available. For more information about our data policy, please see <http://journals.plos.org/plosone/s/data-availability>.

**Ans: Done.**

"Upon re-submitting your revised manuscript, please upload your study’s minimal underlying data set as either Supporting Information files or to a stable, public repository and include the relevant URLs, DOIs, or accession numbers within your revised cover letter. For a list of acceptable repositories, please see <http://journals.plos.org/plosone/s/data-availability#loc-recommended-repositories>. Any potentially identifying patient information must be fully anonymized.

Important: If there are ethical or legal restrictions to sharing your data publicly, please explain these restrictions in detail. Please see our guidelines for more information on what we consider unacceptable restrictions to publicly sharing data: <http://journals.plos.org/plosone/s/data-availability#loc-unacceptable-data-access-restrictions>. Note that it is not acceptable for the authors to be the sole named individuals responsible for ensuring data access.

We will update your Data Availability statement to reflect the information you provide in your cover letter.

3. We notice that your supplementary [figures/tables] are included in the manuscript file. Please remove them and upload them with the file type 'Supporting Information'. Please ensure that each Supporting Information file has a legend listed in the manuscript after the references list.

**Ans: Done.**

4. Please include captions for your Supporting Information files at the end of your manuscript, and update any in-text citations to match accordingly. Please see our Supporting Information guidelines for more information: <http://journals.plos.org/plosone/s/supporting-information>.

**Ans: Done.**
